# Integrated Governance Mechanisms for Empowerment and Resilience in International Food Value Chains

**DOI:** 10.3390/foods12183395

**Published:** 2023-09-11

**Authors:** Juan Manuel Ramon-Jeronimo, Ana Cruz Gonzalez-Calzadilla, Amparo Graciani-Herrero, Raquel Florez-Lopez

**Affiliations:** Department of Financial Economics and Accounting, University Pablo of Olavide of Seville, Ctra. Utrera, Km. 1, 41013 Sevilla, Spainrflorez@upo.es (R.F.-L.)

Considering the significance of the food sector in recent years, the main objective of this Special Issue is to identify the most appropriate governance mechanisms (formalized and relational) for the management of collaborative networks among the international chain of food companies, which are increasingly subject to greater risks and disruptions. These chains are characterized by a high degree of fragmentation, combined with significant asymmetry of power in favor of large international buyer chains, which impose limitations on other companies since they need to reduce their capacity not only to generate value but also to survive. This dependence increases the risk of opportunistic behavior by the dominant partner, coupled with new uncertainties in the target markets (e.g., Brexit, Russian crisis, COVID-19, etc.). To face these challenges, companies need governance mechanisms that increase their resilience to disruptive environments, enabling their empowerment as protection against abuses of power and facilitating the establishment of fair and sustainable value generation and capture processes among all stakeholders.

The global food system faces numerous challenges, including weather disasters, price movements, policy changes, or vulnerabilities due to market shocks [1,2], some of which have been exacerbated by recent international disruptions. To address these complex issues, there is a need to incorporate integrated governance mechanisms that align and coordinate efforts. Such a holistic approach is believed to strengthen food value chains and enable a more comprehensive response to challenges.

In the last few years, the world has suffered some remarkable events that have had a direct effect on international food value chains. Considering this context, in this Special Issue, Ballesteros-Bejarano et al. [3] explore how the COVID-19 pandemic has affected the international operations of Spanish food chains, concluding that even if internationalization is their best opportunity for expansion, it requires a strengthened, flexible response system. In another contribution, Jagtap et al. [4] present the implications of the Russia–Ukraine conflict on global food supply chains, recognizing its impact on the resilience of these chains and providing some strategies to mitigate it.

Natural disasters may also challenge the stability of food chains and trade all around the globe by affecting production levels, which triggers market fluctuations [2]. In line with this fact, it is important to bear in mind the criticality of food supply for survival in times of emergencies [2,5]. Liu et al. [6] emphasize the need to address the food supply chain from a multidisciplinary perspective that ensures appropriate coverage and effective solutions. The integration of different mechanisms can strengthen the capabilities of governance while also ensuring its efficient interaction with the various sources of information [7]. This approach also guarantees that the interaction of management practices with other elements can, by itself, enhance productivity and, therefore, resilience [8].

Furthermore, international food value chains are often characterized by significant power asymmetries between and within countries; for this reason, inclusive cooperation mechanisms need to be upheld [5]. The integration of governance may enhance the development of industries and their competitiveness, as well as drive moderate price movements [1]. Similarly, some specific branches of the food sector, such as the healthy food branch, experience infrastructure gaps and positional difficulties that highlight their need for empowerment [9]. Given the identified spillover effect of price changes in upstream supply chains [1], the empowerment of food value chains could contribute to their resilience against unstable situations.

Food value chains have been experiencing constant global growth [3]. However, internationalization has never been easy for companies [7] as it requires an important investment and is associated with several risks that can mitigate the benefit of internationalization [10].

One of the main risks for food value chains is the lack of food raw materials or finished food products [4]. When this occurs, companies need to have the flexibility and the ability to be resilient to change their strategy to a new one in light of the new challenges [3]. 

At the same time, specific studies in this Special Issue are devoted to analyzing mechanisms that enable companies to face food environment risks such as the Russia–Ukraine conflict, extreme weather disasters, or the COVID-19 pandemic. 

Concerning the mechanisms that companies can implement to respond to risks in international food value chains, studies suggest (i) finding alternative food supply partners; (ii) improving the flexibility of transport logistics regarding global food supply chains; (iii) establishing a policy for maintaining good stock levels, at least, for critical food commodities; and (iv) designing a strategy for workforce safety [4].

In this sense, this Special Issue is devoted to the analysis of integrated governance mechanisms for empowerment and resilience in international food value chains as all the published papers in this collection address all these aspects, thus providing further insights into the mechanisms that companies can put in practice for empowerment and resilience.
